# Sumoylation of cyclin and its therapeutic potential for cancer

**DOI:** 10.1080/19768354.2025.2598975

**Published:** 2025-12-06

**Authors:** Hong-Yeoul Ryu, Mark Hochstrasser

**Affiliations:** a KNU G-LAMP Project Group, KNU Institute of Basic Sciences, School of Life Sciences, BK21 FOUR KNU Creative BioResearch Group, College of Natural Sciences, Kyungpook National University, Daegu, 41566, South Korea; b Department of Molecular Biophysics & Biochemistry, Yale University, New Haven, CT, 06520, USA

**Keywords:** Cyclin D, cyclin E, CDK, sumoylation, cancer

## Abstract

The precision of the cell cycle is essential for organismal development, tissue homeostasis, and the prevention of malignancies. Cyclins and cyclin-dependent kinases (CDKs) play pivotal roles in regulating cell cycle progression. Recent studies have underscored the importance of post-translational modifications, particularly sumoylation, in modulating the functions of cyclins. Sumoylation profoundly influences the stability, localization, and activity of cyclins D and E, which are crucial for the G1/S transition and DNA replication. Dysregulation of these processes is a hallmark of various cancers, where aberrant sumoylation enhances the oncogenic potential of cyclins. This review examines how sumoylation governs cyclin dynamics, maintains cell division fidelity, and contributes to cancer progression. Moreover, advances in targeting the SUMO pathway offer new therapeutic opportunities for treating cyclin-related malignancies, positioning sumoylation-based strategies as promising tools in precision medicine. Gaining a deeper understanding of how sumoylation regulates cyclins may ultimately transform therapeutic approaches for cyclin-dependent diseases.

## Introduction

The cell cycle is an intricate process that governs cell growth, DNA replication, and division, ensuring the faithful transmission of genetic material to daughter cells (Schafer 1998). Precise regulation of the cell cycle is essential for maintaining genomic stability, and its dysregulation is a hallmark of many diseases, notably cancer (Schafer 1998; Jeong et al. 2025; Yoon et al. 2025). Central to the control of this process is a family of proteins known as cyclins, along with their partner enzymes, cyclin-dependent kinases (CDKs) (Hong et al. 2024; Pellarin et al. 2025).

Cyclins are a diverse group of regulatory proteins characterized by their ability to oscillate in abundance during specific phases of the cell cycle (Pellarin et al. 2025). These fluctuations occur through tightly controlled mechanisms involving gene transcription, protein synthesis, and proteolytic degradation. The cyclical nature of cyclin expression acts as a molecular timer, ensuring that cell cycle events occur in the proper sequential order. Cyclins function primarily by binding to specific CDKs, forming active kinase complexes that phosphorylate target substrates, thereby driving progression through various cell cycle phases.

Different cyclins are specialized for different phases of the cell cycle (Martinez-Alonso and Malumbres 2020). D-type cyclins (cyclins D1, D2, and D3) are predominantly involved in the early G1 phase, responding to extracellular signals to promote cell cycle entry from a quiescent state (G0). E-type cyclins (cyclins E1 and E2) are essential for the G1/S transition, driving the initiation of DNA replication. A-type cyclins (cyclins A1 and A2) function during S phase and G2, facilitating DNA synthesis and preparation for mitosis, while B-type cyclins (cyclins B1 and B2) are crucial for the G2/M transition, regulating mitotic entry and chromosome segregation. This stratification of cyclin functions underscores their importance in orchestrating the precise choreography of cell division.

The timely degradation of cyclins, especially at key transitional points between cell cycle phases, is essential for preventing abnormal progression and maintaining cellular homeostasis (Malumbres and Barbacid 2009; Pellarin et al. 2025). Disruptions in cyclin expression or activity through mutations or overexpression have been linked to various human diseases, most notably cancer, where unchecked cyclin activity promotes uncontrolled cellular proliferation. As central regulators of cell cycle progression, cyclins have become prominent targets for therapeutic intervention (Musgrove et al. 2011; Wang et al. 2019).

In addition to their characteristic oscillatory expression, cyclins are regulated at multiple levels, including transcriptional control, post-translational modification (PTM), and targeted degradation via the ubiquitin-proteasome system (Minshull et al. 1989; Glotzer et al. 1991). Among these mechanisms, various studies have identified the attachment of the small ubiquitin-like modifier (SUMO) as a key PTM influencing cyclin activity, stability, and interactions (Eifler and Vertegaal 2015). To date, evidence primarily indicates that cyclins D1 and E are subject to sumoylation (Wang et al. 2011; Bonne-Andrea et al. 2013; Lu et al. 2024), although other cyclins may also be regulated in this manner, and ongoing research continues to explore this possibility. This review examines the emerging understanding of how sumoylation influences cyclins, emphasizing its role in fine-tuning cell cycle regulation and its implications for disease development.

## Sumoylation pathway

**Figure 1.**
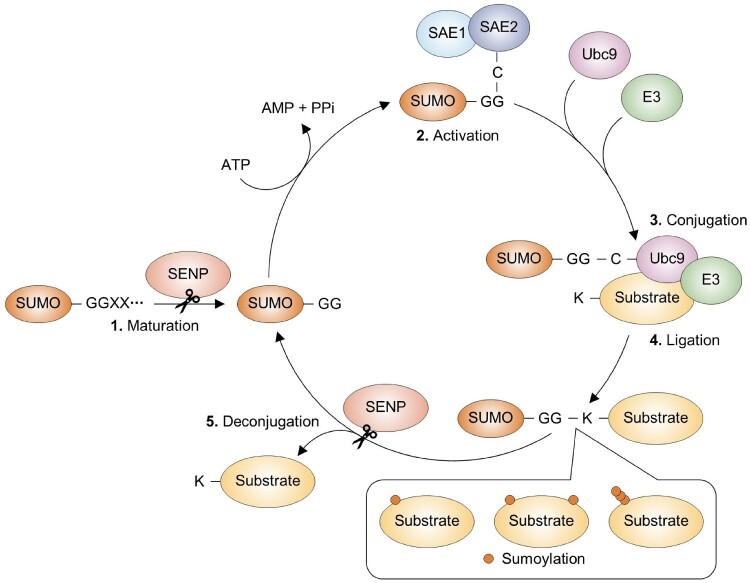
Overview of the sumoylation cycle. 1. Maturation: The precursor SUMO protein is converted into a mature form by the action of SENP, which exposes a C-terminal Gly-Gly (GG) motif. 2. Activation: The mature SUMO is activated in an ATP-dependent manner by a heterodimer of E1 SUMO enzymes, SAE1 and SAE2, which involves interaction with the catalytic cysteine (C) residue on SAE2. 3. Conjugation: The activated SUMO is transferred to the cysteine residue of the E2 SUMO conjugating enzyme Ubc9. 4. Ligation: Assisted by an E3 SUMO ligase, Ubc9 facilitates the covalent attachment of SUMO to lysine (K) residues on target proteins. This process can result in the addition of SUMO to a single lysine (monosumoylation), multiple lysines on the same protein (multisumoylation), or onto another SUMO molecule to form a chain (polysumoylation). 5. Deconjugation: SENP enzymes can remove SUMO from substrate proteins or edit SUMO chains, allowing SUMO to be recycled for subsequent conjugation events.

SUMO proteins are highly conserved regulators found in all eukaryotic organisms (Johnson 2004). In humans, five SUMO paralogs have been identified: SUMO-1, SUMO-2, SUMO-3, SUMO-4, and SUMO-5, with SUMO-1, −2, and −3 being the most extensively studied (Huang et al. 2004; Liang et al. 2016). These SUMO proteins are covalently attached to lysine residues on target proteins through a multi-step enzymatic cascade (Johnson 2004) (Figure 1). SUMO proteins are initially synthesized as precursors with C-terminal extensions, which are processed by SUMO-specific proteases known as SENPs or ULPs to produce mature proteins ending with a conserved glycine-glycine motif essential for conjugation (Johnson 2004; Hickey et al. 2012). The conjugation process begins with activation of the SUMO C-terminus by a heterodimeric SUMO-activating enzyme (E1), composed of SAE1/SAE2, which transfers SUMO to the active site cysteine residue of the SUMO E2 conjugating enzyme Ubc9. Subsequently, various E3 ligases facilitate the transfer of SUMO from Ubc9 to specific lysine residues on target proteins, often within a conserved motif, ΨKX(D/E), where Ψ is a hydrophobic residue, K is the lysine, and X is any amino acid followed by either aspartate or glutamate (Zhao 2018). Sumoylation can occur as a mono-modification or form chain-like polymers (Johnson 2004; Hickey et al. 2012). These modifications are highly dynamic and reversible, with SENPs disassembling SUMO chains and removing SUMO from substrates, allowing for rapid modulation in response to cellular signals (Hickey et al. 2012).

The attachment of SUMO influences numerous cellular functions by altering protein–protein interactions, modulating activity, or inducing conformational changes in its targets. These effects impact critical processes such as transcription regulation, DNA repair, mitochondrial function, ribosome biogenesis, apoptosis, stress responses, and cell cycle progression (Flotho and Melchior 2013; Ryu et al. 2016; Ryu et al. 2019; Ryu et al. 2020; Ryu et al. 2020; Ryu 2022; Jeong et al. 2025).

Recent research emphasizes that sumoylation functions as an active coordinator of key cell cycle transitions rather than serving as a passive modification. Sumoylation targets crucial cell cycle regulators, such as CDKs, cyclins, Aurora-B, retinoblastoma (Rb), topoisomerase II, Nuf2, BubR1, CDCA8, RhoGDIα, and FoxM1, modulating their function in concert with additional post-translational modifications to ensure accurate regulation of cell cycle progression (Wang et al. 2011; Bonne-Andrea et al. 2013; Eifler and Vertegaal 2015; Meng et al. 2016; Chen et al. 2024; Lu et al. 2024). For example, the activity of Aurora-B kinase during oocyte meiosis is regulated by SUMO-2/3 modification, which is essential for proper spindle assembly and chromosome alignment, and mutations that prevent this SUMO-2/3 conjugation to Aurora-B disrupt these processes and impair meiotic progression (Chen et al. 2024). In addition, Rb is sumoylated at the early G1 phase, which promotes its phosphorylation by recruiting CDK2 through a SUMO-interaction motif (SIM), leading to Rb hyperphosphorylation and cell cycle progression, while loss of sumoylation reduces CDK2 binding, phosphorylation, and cell proliferation (Meng et al. 2016). Parallel studies have uncovered SUMO-mediated degradation systems that interface with the ubiquitin – proteasome network, such as the SUMO1 – PSME3–20S proteasome axis, which ensures timely clearance of cell cycle transcription factors like CP2c to maintain G1/S progression (Son et al. 2023).

These SUMO-mediated events illustrate that SUMO-dependent cell cycle control is finely tuned by coordinated conjugation and deconjugation. SENPs play central roles in maintaining this balance by regulating SUMO turnover on mitotic substrates. Among them, the SENP2 localization to kinetochores and spindle structures is essential for accurate mitotic progression, and its loss results in spindle defects, chromosome misalignment, and premature epithelial differentiation (Galan-Vidal et al. 2024). Notably, reduced SENP2 expression has been associated with elevated immuno-checkpoint biomarker PD-L1 levels in aggressive cancers, highlighting its dual role as a regulator of epithelial mitosis and as a potential biomarker for tumor progression (Galan-Vidal et al. 2024).

Together, these findings support a refined model in which sumoylation functions as a molecular rheostat that integrates checkpoint signaling, cyclin – CDK activity, and proteolytic degradation to ensure precise control of cell division.

## Sumoylation of cyclin D1

**Figure 2.**
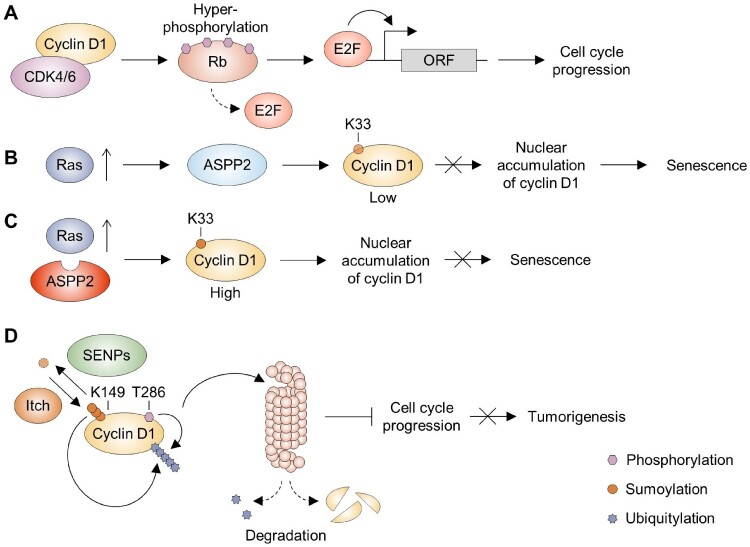
Sumoylation of cyclin D1 in RAS-induced senescence and tumorigenesis. (A) Cyclin D1, in complex with CDK4/6, promotes the hyperphosphorylation of Rb, leading to the release of E2F and activation of replication gene expression, thereby advancing the cell cycle. (B) Ras oncogene-induced senescence is mediated by ASPP2, which prevents the sumoylation of cyclin D1, thereby reducing its nuclear accumulation. (C) Cyclin D1 becomes highly sumoylated, particularly at K33, in cells with activated RAS and ASPP2 mutation, which results in increased nuclear localization of cyclin D1 and subsequently bypasses Ras-induced senescence. (D) Phosphorylation at T286 and Itch SUMO E3 ligase-mediated polysumoylation at K149 of cyclin D1 promote its degradation via the ubiquitin-proteasome pathway, thereby preventing tumorigenesis by inhibiting cell cycle progression.

Cyclin D1 plays a critical role in normal cell cycle progression, particularly during the G1/S transition by forming complexes with CDK4/6 to promote cellular proliferation (Martinez-Alonso and Malumbres 2020). However, dysregulation of cyclin D1 is frequently observed in human cancers, with gene amplification and overexpression documented in approximately 50% of breast and esophageal cancers (Barnes and Gillett 1998; Shamma et al. 2000). Importantly, nuclear accumulation of cyclin D1 is essential for its oncogenic activity because it enables phosphorylation of the Rb protein, which releases E2F transcription factors and activates the expression of replication genes during S phase, thereby promoting cell cycle progression (Diehl et al. 1998; Alt et al. 2000) (Figure 2(A)). Mutant forms of cyclin D1, which are defective in nuclear export and degradation, can drive cellular transformation and tumor formation. For example, the T286A mutant, which resists phosphorylation-dependent nuclear export, induces cellular transformation *in vitro* and tumor development *in vivo*, including B-cell lymphomas and mammary adenocarcinomas (Alt et al. 2000; Gladden et al. 2006). These findings highlight the significance of subcellular localization and post-translational regulation in controlling the oncogenic potential of cyclin D1.

Beyond phosphorylation-dependent mechanisms, other PTMs such as sumoylation have been implicated in regulating nuclear localization and oncogenic capacity of cyclin D1 (Wang et al. 2011). Ras oncogene-induced senescence can be modulated by apoptosis-stimulating protein of p53 (ASPP) 2, which regulates the apoptotic function of p53 and its family members p63 and p73 (Samuels-Lev et al. 2001; Wang et al. 2011) (Figure 2(B)). In cells harboring ASPP2 mutants lacking exon 3, sumoylation was identified as a novel regulatory process promoting nuclear localization of cyclin D1 (Wang et al. 2011) (Figure 2(C)). Multiple cyclin D1 lysine residues, including K33, have been characterized as important sites for sumoylation, with modification at K33 playing a particularly prominent role (Wang et al. 2011). Nuclear localization of cyclin D1 stimulates its oncogenic functions both *in vitro* and *in vivo*, as nuclear cyclin D1 has been shown to bypass Ras-induced senescence (Wang et al. 2011). These findings suggest that sumoylation, especially at K33, acts as a key regulatory switch that enhances cyclin D1’s oncogenic activity by promoting its nuclear accumulation, further emphasizing the importance of PTMs in cancer progression.

Furthermore, the steady-state levels of cyclin D1 are tightly controlled by proteasome-mediated degradation mechanisms involving both phosphorylation and sumoylation (Diehl et al. 1997; Guo et al. 2005; Lu et al. 2024) (Figure 2(D)). Phosphorylation at T286 of cyclin D1 facilitates its recognition by the ubiquitin-proteasome system, leading to its degradation and preventing excessive accumulation (Diehl et al. 1997; Guo et al. 2005). Simultaneously, polysumoylation at K149 of cyclin D1 enhances its recognition by the specific SUMO E3 ligase Itch, facilitating ubiquitylation and subsequent degradation (Lu et al. 2024). Sumoylation and ubiquitination are known to compete for modification on the same lysine residues in many proteins (Ryu and Hochstrasser 2021), although such direct competition has not been demonstrated specifically for cyclins. Disruption of these degradation pathways through mutations at either the phosphorylation or sumoylation sites leads to persistent cyclin D1 stabilization, promoting B lymphocyte transformation and a lymphoma-like phenotype reminiscent of mantle cell lymphoma (MCL) (Lu et al. 2024). Notably, pharmacological approaches such as arsenic trioxide, a front-line treatment for acute promyelocytic leukemia (Schick et al. 2022), increases sumoylation-mediated degradation of cyclin D1 by inhibiting SENPs, resulting in apoptosis of MCL cells (Lu et al. 2024). Overall, these insights underscore how a delicate balance of phosphorylation – and sumoylation-dependent degradation pathways is essential to prevent cyclin D1-driven tumorigenesis, and their dysregulation contributes to the development of lymphomas, providing potential targets for therapeutic intervention.

## Sumoylation of cyclin E

The intricate role of sumoylation in DNA replication and repair processes is exemplified by the modification of proliferating cell nuclear antigen (PCNA) with SUMO at DNA replication forks (Hoege et al. 2002; Leach and Michael 2005; Moldovan et al. 2012). While PCNA (yeast Pol30) sumoylation can be difficult to detect due to the low proportion of modified protein, and is not essential during normal yeast growth, it becomes significant upon DNA damage. Such damage stimulates sumoylation of PCNA which in turn leads to the recruitment of proteins such as Srs2, which is a DNA helicase involved in DNA repair, to stalled replication forks (Pfander et al. 2005).

**Figure 3.**
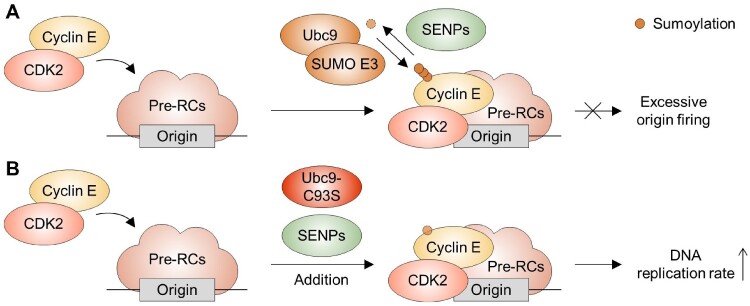
Sumoylation of cyclin E in replication origin firing. (A) Cyclin E, in complex with CDK2, is recruited to pre-replicative complexes (pre-RCs) at replication origins, where it undergoes sumoylation. This dynamic SUMO modification is regulated by the antagonistic actions of Ubc9/SUMO E3 ligases and SENPs, and serves to limit excessive origin firing. (B) The addition of Ubc9-C93S and SENPs to prevent sumoylation during the DNA replication reaction resulted in a decrease in the sumoylation of cyclin E and an increase in the replication rate.

Beyond PCNA, numerous DNA replication and repair proteins are sumoylated especially under stress conditions, underscoring the critical function of this modification in genomic stability (Golebiowski et al. 2009; Bruderer et al. 2011; Cremona et al. 2012). Among these, cyclin E emerges as a pivotal SUMO pathway target, particularly marked by its extensive SUMO2/3 conjugation on chromatin (Bonne-Andrea et al. 2013) (Figure 3). Approximately 50% of chromatin-bound cyclin E is sumoylated following the recruitment of cyclin E–Cdk2 complexes to pre-replicative complexes (pre-RCs), highlighting its central role in regulating replication origin firing during early S phase (Bonne-Andrea et al. 2013). This SUMO2/3 modification of cyclin E is dynamically regulated by SENPs and plays a crucial role in preventing excessive origin firing, independent of Cdk2 activity (Bonne-Andrea et al. 2013). Addition of a dominant-negative mutant of Ubc9 (Ubc9-C93S) (Azuma et al. 2003) and SENP, both of which block sumoylation during replication, significantly reduces sumoylated cyclin E and greatly increases the rate of replication. While cyclin E is a primary target, other replication proteins may also be sumoylated, suggesting a broader role for sumoylation in the nuanced regulation of origin firing.

## SUMO-mediated regulation of cyclin

**Figure 4.**
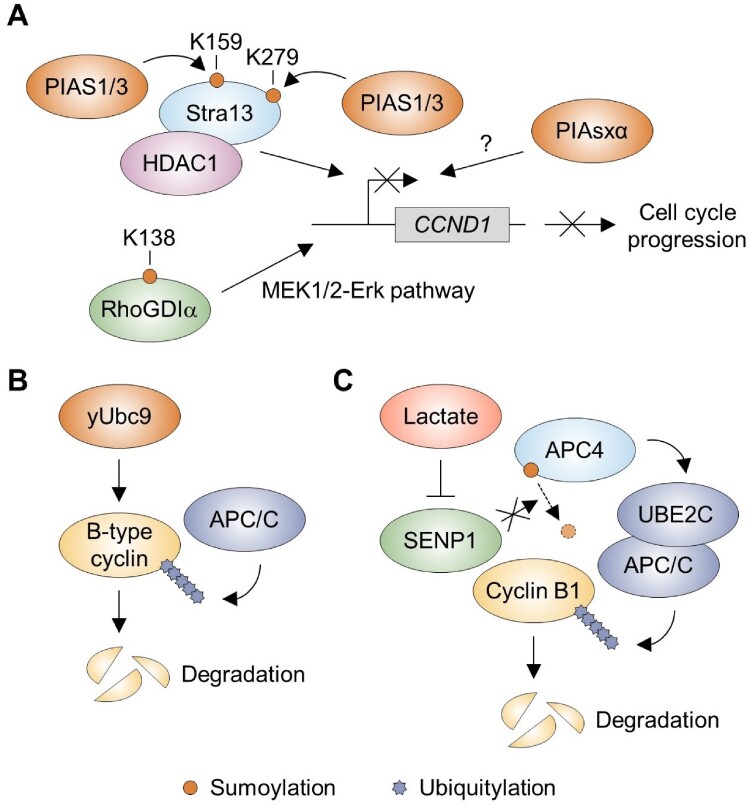
Sumoylation-mediated indirect regulation of cyclin. (A) Sumoylation at K159 and K279 of Stra13 by the PIAS1/3 SUMO E3 ligase, which interacts with HDAC1, activation of the MEK1/2-Erk pathway through sumoylation at K138 of RhoGDIα, and the PIASxα SUMO E3 ligase together suppress cyclin D1 (*CCND1*) transcription, resulting in the blockade of cell cycle progression. (B) In yeast, Ubc9 E2 enzyme facilitates the APC/C-mediated ubiquitylation and degradation of B-type cyclins. (C) In humans, lactate inhibits the SENP1, resulting in stabilized sumoylation of the APC4. This promotes the UBE2C binding to the APC/C, facilitating the targeted degradation of cyclin B1.

Sumoylation can exert significant control over cyclins through indirect pathways, notably by influencing transcription factors that regulate the cyclin genes (Figure 4A). For instance, Stra13, a member of the bHLH-O repressor subfamily that interacts with the co-repressor HDAC1 (Sun and Taneja 2000), is sumoylated at K159 and K279 (Wang et al. 2012). These modifications, catalyzed by the SUMO E3 ligases PIAS1 and PIAS3, enhance Stra13's repressor activity (Wang et al. 2012). Mutations at these residues impair Stra13's role in repressing expression of the cyclin D1 gene (*CCND1*) and diminish its function as a growth suppressor, partly due to reduced association with HDAC1, which is crucial for cell cycle regulation (Wang et al. 2012). Additionally, RhoGDIα, which governs Rho family GTPases (Garcia-Mata et al. 2011), is sumoylated at K138, which is critical for its role in inhibiting cancer cell motility and invasion (Cao et al. 2014). A K138R mutation diminishes RhoGDIα's capacity to repress gene expression of the cyclin D1 gene and induce G0/G1 cell cycle arrest, and sumoylation at K138 is required for RhoGDIα repression of the MEK1/2/Erk pathway (Cao et al. 2014). Furthermore, protein inhibitor of activated STATxα (PIAsxα), a SUMO E3 ligase (Wang et al. 2014), is linked to several types of cancer, including pancreatic cancer, lung cancer, colorectal cancer, and hepatocellular carcinoma (Okumura et al. 2010; Liu et al. 2011; Li et al. 2013). It suppresses expression of cyclin D1 and D3, contributing to the inhibition of osteosarcoma cell development (Wang et al. 2016).

Cyclins are tightly regulated by proteolytic degradation. In yeast, Ubc9, the SUMO E2 conjugating enzyme, has been shown to be essential for progression through mitosis by promoting the degradation of the B-type cyclins such as Clb2 and Clb5 (Seufert et al. 1995) (Figure 4(B)). Both Ubc9 and Smt3, the yeast SUMO protein, are required for the anaphase-promoting complex/cyclosome (APC/C)-mediated degradation of key mitotic regulators such as Pds1 securin and Clb2 (Dieckhoff et al. 2004). In humans, lactate inhibits SENP1 by forming an inhibitory complex with zinc at its active site, rapidly stabilizing the sumoylation of APC4, which drives binding of the UBE2C ubiquitin E2 enzyme to APC/C (Liu et al. 2023). This remodeling of the APC/C facilitates the targeted degradation of cyclin B1 and securin, thereby ensuring efficient mitotic exit (Liu et al. 2023) (Figure 4(C)). These findings, along with examples of transcriptional regulation, underscore the versatile role of sumoylation in indirectly modulating cyclin levels, thereby maintaining proper cell cycle control and influencing cellular growth.

SUMO-targeted ubiquitin ligases (STUbLs) are specialized E3 ubiquitin ligases that recognize sumoylated proteins and target them for ubiquitination, often leading to proteasomal degradation (Sriramachandran and Dohmen 2014). Well-known human STUbLs include RNF4 and Arkadia/RNF111, which bind sumoylated substrates via SIMs to control protein stability and function (Sriramachandran and Dohmen 2014). However, direct evidence showing cyclins as STUbL targets remains limited or indirect. Current studies emphasize STUbL regulation of DNA repair factors, transcription regulators, and chromatin-associated proteins rather than cyclins explicitly. The degradation of cyclins is controlled primarily by canonical ubiquitin ligases such as SCF-FBXW7, rather than by known STUbLs (Zhang and Koepp 2006; Hao et al. 2007). Therefore, while STUbLs broadly regulate sumoylated proteins critical for genome stability and cell cycle progression, direct STUbL targeting of cyclins has not yet been conclusively demonstrated.

## Recent advances in cyclin-targeted therapies for cancer

In recent years, targeting cyclins and their associated CDKs has become a cornerstone of therapeutic strategies, particularly in oncology. Cyclin D forms complexes with CDK4 or CDK6, playing a crucial role in initiating cell cycle progression (Schafer 1998). Its hyperactivation, often resulting from gene amplification or point mutations, drives oncogenic cell proliferation in various cancers (Sherr et al. 2016; Wei et al. 2024). Targeting CDK4 and CDK6 with specific inhibitors has emerged as an important strategy in cancer therapy, particularly in hormone receptor-positive breast cancer, where agents such as palbociclib (PD-0332991), ribociclib (LEE011), and abemaciclib (LY2835219) have demonstrated significant clinical efficacy (Finn et al. 2016; Tripathy et al. 2017; Ademuyiwa et al. 2023; Fung and Blair 2023). These drugs function by selectively inhibiting the kinase activity of CDK4 and CDK6, preventing phosphorylation of the Rb protein, and thereby halting cell cycle progression in G1. Despite their success, response rates vary significantly among patients, and in tumors heavily dependent on cyclin D1 dysregulation, the effectiveness of CDK4 and CDK6 inhibitors is often limited (Herrera-Abreu et al. 2016; Wander et al. 2020).

**Figure 5.**
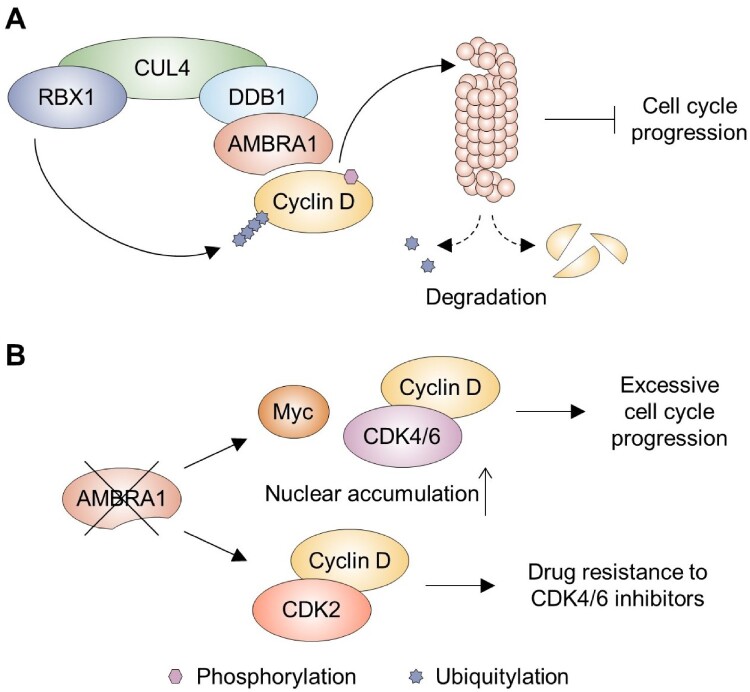
Regulation of cyclin D by CRL4^AMBRA1^ complex. (A) The CRL4^AMBRA1^ complex, which includes CUL4, RBX1, DDB1, and AMBRA1, facilitates the poly-ubiquitylation of the phosphorylated form of cyclin D, targeting it for degradation via the ubiquitin-proteasome pathway. This action inhibits cell cycle progression. (B) When AMBRA1 is lost or downregulated, cyclin D and MYC accumulate in the nucleus, leading to excessive cell proliferation. Additionally, cyclin D can aberrantly bind to CDK2, resulting in drug resistance to CDK4/6 inhibitors.

Recent research has shed light on the detailed molecular process responsible for cyclin D degradation (Chaikovsky et al. 2021; Maiani et al. 2021; Simoneschi et al. 2021). Central to this process is the CRL4^AMBRA1^ complex, an E3 ubiquitin ligase comprised of a scaffold protein Cullin4 (CUL4), a catalytic RING box protein 1 (RBX1), damage-specific DNA binding protein 1 (DDB1), and activating molecule in beclin-1-regulated autophagy (AMBRA1) (Simoneschi et al. 2021) (Figure 5A). This complex specifically ubiquitinates the phosphorylated form of cyclin D, marking it for degradation by the proteasome (Chaikovsky et al. 2021). When AMBRA1 is lost or downregulated, cyclin D and MYC family proteins tend to accumulate, leading to excessive cell proliferation and increased risk of tumor formation, as seen in cancers such as lung adenocarcinoma and lymphoma (Chaikovsky et al. 2021; Maiani et al. 2021) (Figure 5B). Moreover, in cells with reduced AMBRA1, cyclin D can aberrantly bind to CDK2, bypassing the inhibitory effects of CDK4 and CDK6 inhibitors and thus conferring drug resistance (Chaikovsky et al. 2021; Simoneschi et al. 2021). These findings highlight the importance of proteolytic pathways in regulating cyclin D and offer promising avenues for designing more effective, targeted cancer therapies.

Aside from cyclin D, cyclin E, which interacts with CDK2, is also strongly implicated in various cancers. A wide array of strategies has been employed to inhibit cyclin E activity or expression. For example, the quinolone-sulfonyl compound VR23 promotes ubiquitination and proteasomal degradation of cyclin E, thereby halting tumor cell proliferation (Pundir et al. 2015). Alternately, the phenylpropanoid-based sulfonamides, Ganoderiol F or tehranolide, have been shown to induce G0/G1 arrest via downregulation of both cyclins D1 and E expression. These examples highlight ongoing efforts to therapeutically target cyclin E-driven pathways in cancer treatment (Noori and Hassan 2012; Azevedo-Barbosa et al. 2019; Li et al. 2019).

Interestingly, MicroRNAs such as miR-195 act as tumor suppressors by directly targeting *CCNE1*, which encodes Cyclin E1, thereby reversing chemoresistance in glioma (Wang et al. 2019). Furthermore, human antigen R (HuR), an RNA-binding protein whose overexpression in human cancer correlates with aggressive disease, drug resistance, and poor prognosis, contributes to cyclin E deregulation in cancer cells. CMLD-2, a small-molecule inhibitor targeting HuR, emerges as a promising therapeutic agent for targeting this pathway (Guo and Hartley 2006; Muralidharan et al. 2017). Collectively, these advances underscore the potential of diverse molecular and pharmacological interventions to selectively inhibit cyclin E activity and improve outcomes in malignancies heavily reliant on this pathway.

Importantly, the therapeutic potential of cyclin modulation extends beyond oncology. Evidence from neurodegenerative disease models suggests that regulating CDK and cyclin activity could offer neuroprotective effects, preventing aberrant cell cycle re-entry in neurons, a process implicated in neurodegeneration. For instance, specific CDK inhibitors are being explored for their capacity to mitigate neuronal death and promote regeneration in models of Alzheimer’s disease (Malhotra et al. 2021). Additionally, fine-tuning cyclin activity holds promise for treating age-related proliferative disorders and restoring tissue homeostasis.

## Conclusion

The targeting of cyclins, particularly cyclin D and cyclin E, has become a major focus in the development of cancer therapeutics, with several drugs already demonstrating clinical efficacy (Musgrove et al. 2011). As our understanding of human cell cycle regulation deepens, it is increasingly evident that PTMs, such as sumoylation, offer promising new targets for intervention. Recent advances highlight the potential of manipulating SUMO pathways by either selectively inhibiting or enhancing the sumoylation of targets to modulate their stability, activity, and protein interactions (Kukkula et al. 2021; Park et al. 2021; Jeoung et al. 2022). Such approaches could provide a more refined and specific strategy compared to conventional drugs that broadly target cyclins or CDKs, potentially reducing side effects and improving safety profiles.

However, given that sumoylation modifies thousands of proteins involved in diverse cellular processes, systemic modulation of the SUMO pathway carries a risk of significant off-target toxicity (Kukkula et al. 2021). Therefore, future therapeutic strategies should emphasize precision targeting. One promising approach is to engineer bifunctional molecules in which a targeting moiety, including a small-molecule ligand, nucleic acid aptamer, peptide, or engineered protein binder (e.g. nanobody or monobody) (Sandhof et al. 2025), with high specificity for a protein of interest, such as a cyclin or CDK, is chemically linked to a SUMO E3 ligase or SENP. This design enables the direct recruitment of SUMO-conjugating or deconjugating activity to the target protein, allowing for selective modulation of its sumoylation status without broadly affecting the global SUMO landscape. Such strategies leverage the molecular recognition capabilities of diverse targeting platforms and can be further refined through rational design, high-throughput screening, and synthetic biology appr\oaches to maximize specificity and efficacy. These advances open the door to highly targeted, reversible, and safer manipulation of sumoylation for therapeutic benefit. Ultimately, harnessing the nuanced regulation of cyclins via sumoylation represents a promising frontier for advancing precision medicine in oncology and beyond.
